# Quantifying the protective capacity of mangroves from storm surges in coastal Bangladesh

**DOI:** 10.1371/journal.pone.0214079

**Published:** 2019-03-21

**Authors:** Susmita Dasgupta, Md. Saiful Islam, Mainul Huq, Zahirul Huque Khan, Md. Raqubul Hasib

**Affiliations:** 1 Development Research Group, World Bank, Washington, DC, United States of America; 2 Coast, Port and Estuary Management Division, Institute of Water Modeling, Dhaka, Bangladesh; 3 World Bank, Dhaka, Bangladesh; Tanzania Fisheries Research Institute, UNITED REPUBLIC OF TANZANIA

## Abstract

Mangroves are an important ecosystem-based protection against cyclonic storm surge. As the surge moves through the mangrove forest, the tree roots, trunks, and leaves obstruct the flow of water. Damage to adjacent coastal lands is attenuated mainly by reducing (i) surge height, which determines the area and depth of inundation and (ii) water flow velocity. But the extent of mangrove protection depends on the density of tree plantings and the diameter of trunks and roots, along with an array of other forest characteristics (e.g., floor shape, bathymetry, spectral features of waves, and tidal stage at which waves enter the forest). Making efficient use of mangroves’ protective capacity has been hindered by a lack of location-specific information. This study helps to fill that gap by estimating reduction in storm surge height and water flow velocity from mangroves at selected sites in cyclone-prone, coastal Bangladesh. A hydrodynamic model for the Bay of Bengal, based on the MIKE21FM system, was run multiple times to simulate the surge of cyclone Sidr (2007) at the Barisal coast. Estimates of surge height and water flow velocity were recorded first without mangroves and then with mangroves of various forest widths and planting densities, including specific information on local topography, bathymetry, and Manning’s coefficients estimated from species’ root and trunk systems. The results show a significant reduction in water flow velocity (29–92%) and a modest reduction in surge height (4–16.5 cm). These findings suggest that healthy mangroves can contribute to significant savings in rehabilitation and maintenance costs by protecting embankments from breaching, toe-erosion, and other damage.

## Introduction

Coastal managers increasingly recognize the protective role of mangroves as a disaster risk-reduction tool. This recognition reflects the emphasis that recent scientific literature has placed on the role of mangroves in protecting adjacent coastal lands from the impacts of inundation and erosion, both during natural disasters and through their longer-term influence on coastal dynamics [1–6]. Numerous modeling and mathematical studies have shown that, during cyclones, mangrove forests can attenuate surge height and water flow velocity owing to the matrix of mangrove tree roots, trunks, and leaves obstructing the flow of water through the forest, which creates bed resistance [7–17]. However, most scientific studies conclude that the effective design and management of mangrove forests for providing “natural protection” to coastal communities and assets require additional, location-specific research data on the density and width of tree plantings and the diameter of trunks and roots, along with an array of other information (e.g., floor shape, bathymetry, and spectral features of waves) to define the details and limits of mangroves’ protective function [18–20]. Making efficient use of mangroves’ protective capacity has often been hindered by a lack of location-specific information [4,21]. This study helps to fill that gap by quantifying the protective capacity of mangroves in cyclone-prone, coastal Bangladesh.

In many of the world’s coastal areas, adaptation to climate change will require living with sea-level rise and intensified storm surges. The role of mangroves as protection against coastal hazards—either as stand-alone natural protection or in combination with built infrastructure—has been the focus of considerable attention in recent years, especially in the aftermath of the 2004 Indian Ocean tsunami. Bangladesh—one of the world’s most vulnerable countries to tropical cyclones [22–24]—provides an ideal case for investigating the protective capacity of mangrove forests against cyclonic storm surges as the country has no other natural barriers against this hazard (e.g., sea grass or coral reefs) near its coastline. Between 1960 and 2016, Bangladesh was hit by 23 severe cyclonic storms and 10 cyclonic storms, 26 of which recorded hurricane wind speeds of 118 km per hour. During cyclones, massive storm surges are a major threat to lives and assets in the country’s low-lying coastal districts along the Bay of Bengal. In the past 12 years alone, cyclones Sidr (2007), Aila (2009), and Roanu (2016) induced devastating storm surges, with heights of 1.5–4 m. When storm surges strike densely populated areas during high tide, the impacts are particularly disastrous.

Since the early 1960s, a total of 139 polders, 49 of which are sea-facing, have been constructed in Bangladesh—most of them built during the 1960s and 1970s and in recent years—to protect low-lying coastal areas against mainly tidal floods and salinity intrusion [25]. At present, the heights of these polders are at increasing risk of overtopping, particularly those located in the southwest coastal region, where once infrequent cyclone strikes are on the rise. In response to a growing concern about the potential impact of more frequent cyclones and sea-level rise in a changing climate, the Government of Bangladesh, in collaboration with the World Bank, initiated the Coastal Embankment Improvement Project, which aims to enhance the heights of 17 selected polders in the southwest coastal region.

This study was undertaken to develop the basis for the engineering judgment necessary for designing the forest cover for various foreshore segments of the selected polders. The question was whether mangroves planted in the foreshore area of embankments could play a significant role in protecting against cyclonic storm surges. That is, to what extent could mangroves attenuate surge height—and thus complement the height of built embankments—and reduce water flow velocity during storm surges? To answer these questions, a World Bank research team, in consultation with experts from Bangladesh, conducted storm surge modeling with a hydrodynamic model for the Bay of Bengal, based on the MIKE21FM system. Findings confirm varying levels of protection from mangroves, depending on the selected species, forest width, and planting density.

## Materials and methods

### Study area selection

**Fig 1:**
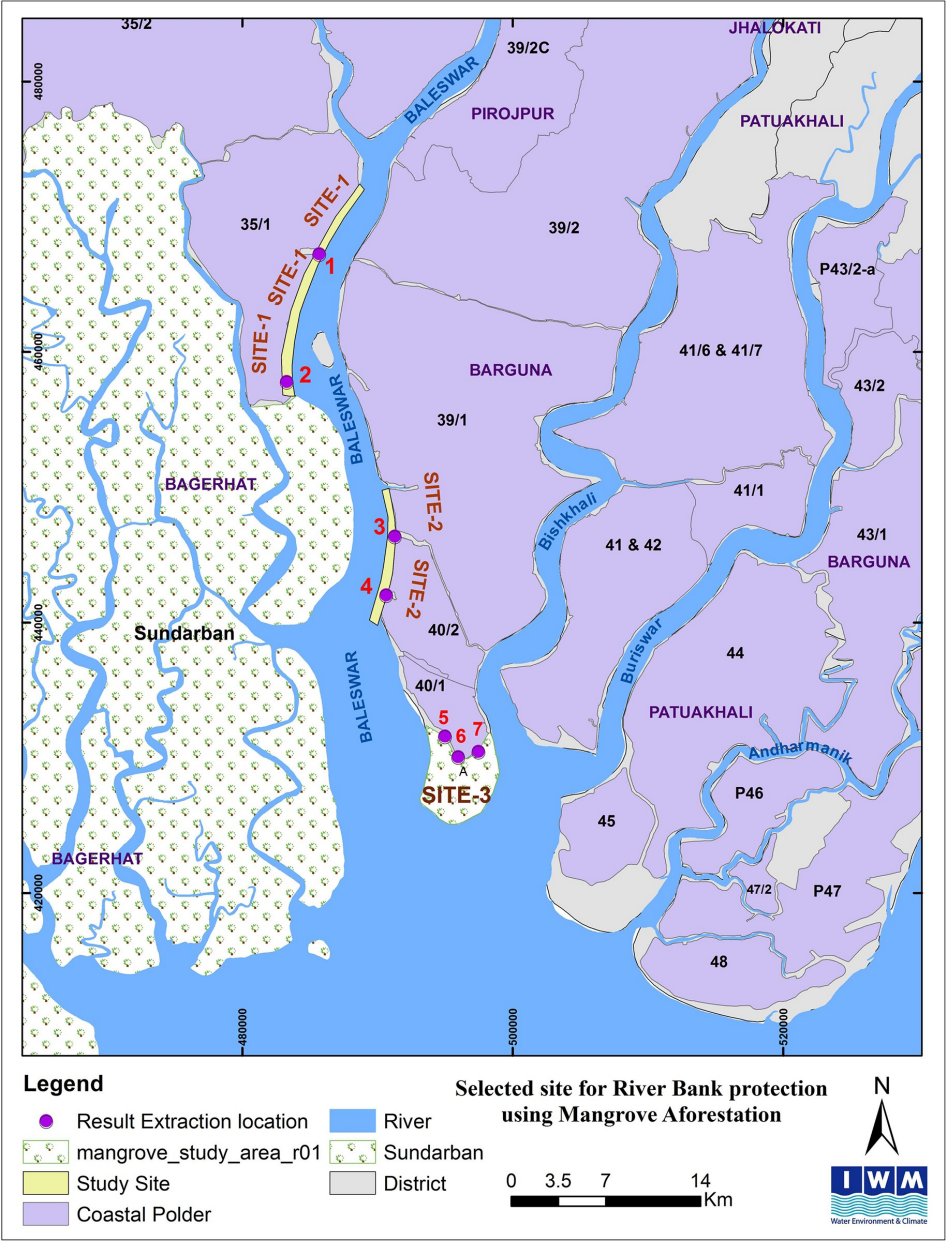
Three study areas, highlighting the seven sites for analysis.

Three study areas (a total of seven sites) with existing polders in Bangladesh’s southwest coastal region were selected for this hydrological analysis (Fig 1). These sites are located in Bagerhat and Barguna districts, where recurrent cyclones in recent years have been cited as a major reason for high incidence of poverty [26,27]. The study was conducted primarily with secondary data from the study areas, which did not include protected areas or private land and did not involve endangered or protected species.

The three areas selected for the analysis are located (i) at the foreshore of polder 35/1, (ii) adjacent to polder 40/2, and (iii) at the southern foreshore of polder 40/1 in the Sundarbans and central coast. Selection of these three areas was based on storm-surge vulnerability and tidal characteristics (e.g., mud flat accretion/erosion and availability of adequate mangrove afforestation area in the foreshore of the coastal polders) (Table 1).

**Table 1:** Key characteristics of the three study areas.

Study area	Administrative district (upazila or sub-upaliza: union)	Location	Mud flat/ tidal feature	Maximum salinity (ppt)	Setback distance of the polder (foreshore)
1. Adjacent to polder 35/1	Bagerhat (Sarankhola: Khontakata, Rayenda, and Dakhin Khali)	River-facing, along the right bank of the Baleswar River	Mud flat erosion	8.25	50–70 m
2. Adjacent to polder 40/2	Barguna (Patharghata: Kanthal Tali and Char Duani)	River-facing, along the left bank of the Baleswar River	Mud flat accretion	10.80	100–150 m
3. Adjacent to polder 40/1	Barguna (Patharghata: Patharghata)	Sea-facing, exposed to the estuary of the Baleswar and Bishkhali Rivers	Mud flat accretion	20.30	2 km

Dasgupta et al. (2015) projected location-specific aquatic salinity in 2050 for southwest coastal Bangladesh, including study areas 1–3, with and without climate change [28]. In a changing climate, salinity in study areas 1 and 2, which are river-facing, is expected to increase due to sea-level rise. Dasgupta, Sobhan, and Wheeler (2017) estimated the salinity tolerance thresholds of mangrove species commonly found in southwest coastal Bangladesh, including study areas 1–3, and projected their expected transition with progressive aquatic salinization [29]. The present research took these findings into account.

### Species selection

Selection of mangrove species was based on high-resolution species distribution maps provided by Bangladesh’s Department of Forests. For each of the seven study sites, the selection of suitable mangrove species took into account tidal characteristics, current and projected future salinity tolerance of the species [28,29], and water salinity [30]. On consultation with ecologists, seven mangrove species likely to survive in the current water salinity were short-listed: *Avicennia officinalis*, *Bruguiera gymnorhiza*, *Ceriops decandra*, *Exoecaria agallocha*, *Heritiera fomes*, *Sonneratia apetala*, and *Xylocarpus granatum*. Subsequent to field visits and focus group discussions with local inhabitants, location-specific mangrove species were selected. *S*. *apetala* and *A*. *officinalis* were chosen for all three study areas, while study area 3 (at the southern foreshore of polder 40/1 in the Sundarbans and central coast) also included *H*. *fomes*, *E*. *agallocha*, and *C*. *decandra*.

### Estimation of Manning’s roughness coefficients

In the absence of age-specific data on young trees during their life cycle and considering that mangrove species require many years to reach maturity (e.g., 25 years for *S*. *apetala* and 30–35 years for *A*. *officinalis*), Manning’s coefficients were estimated for mature trees only. At each of the seven sites in the three study areas, field measurements were taken of mature trees for each selected species. These measurements were for diameter of tree trunks and roots, height of roots from the ground level, and distance between trees (S1 Fig). Also, local experts were consulted on the feasible density and spacing of plantings. For each mangrove species, the Manning’s numbers for different water levels were estimated first for their root systems and then combining with that of the trunk systems for alternative spacing between trees. In the subsequent hydrological analysis, these Manning numbers served as estimates of water-flow resistance from the mangrove trees while passing through the forest.

### Model setup

A hydrodynamic model for the Bay of Bengal was set up and updated with location-specific data to represent the coastal, as well as estuarine, features of the three study areas. The model was upgraded with conversion of structured computational grid (MIKE 21Classic) to a flexible mesh system (MIKE 21FM) to enhance the resolution of the grids around islands and along coastline and other areas of interest. Three open boundaries—two in the north and one in the south—were defined in the model, covering the area from Baruria on the Padma River down to 16° north latitude in the Bay of Bengal (S2 Fig).

To generate the storm surge model, cyclone tracks and cyclonic wind and pressure field information generated from historic cyclone data were added to the hydrodynamic model as inputs of meteorological parameters. Holland Single Vortex theory was applied to generate the cyclonic wind field [31]. The generated cyclonic wind and pressure fields for the cyclone track were then used in the hydrodynamic model to simulate the cyclonic storm surges.

### Cyclone simulations

**Fig 2:**
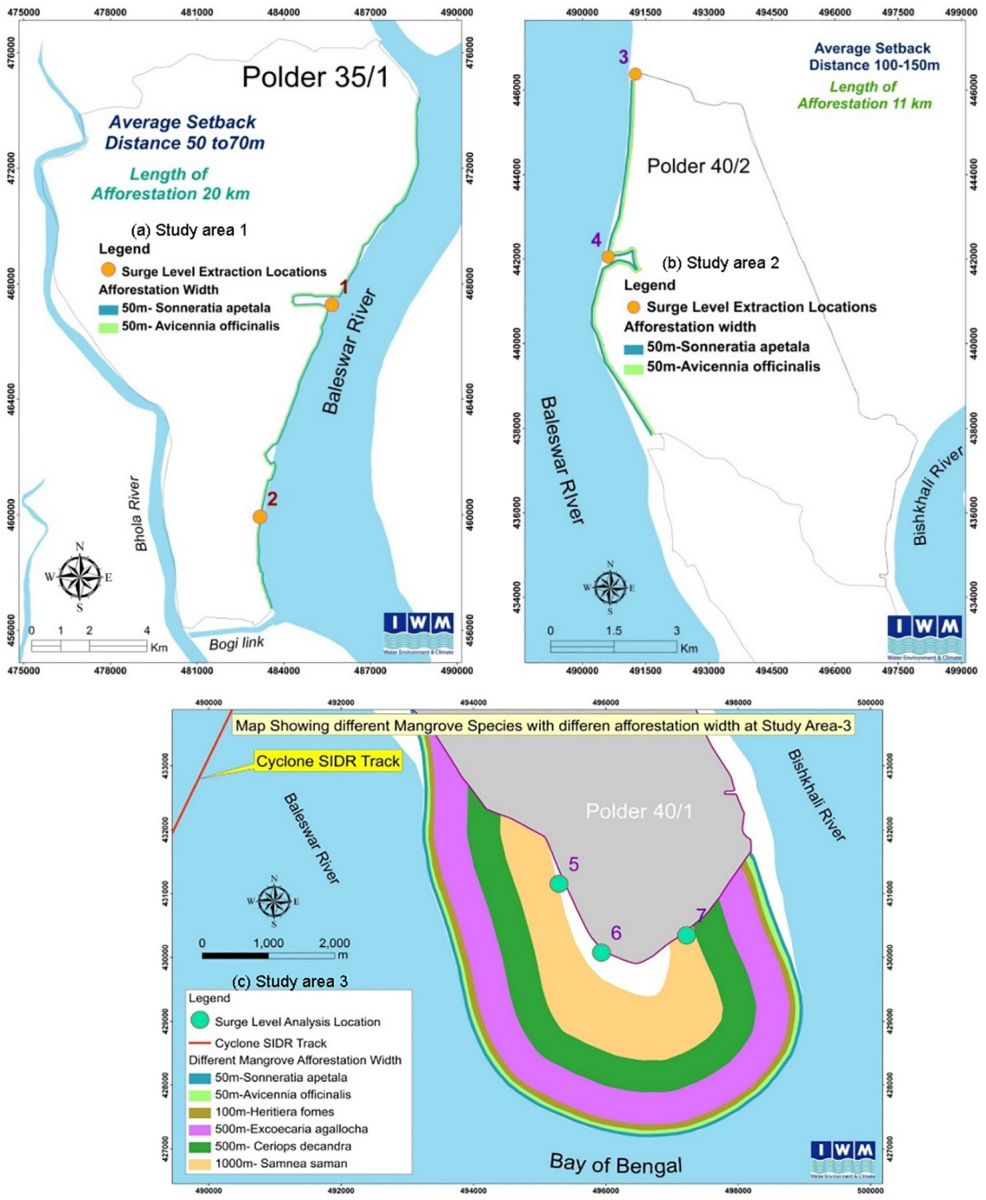
Afforestation area for analysis. (a) Study area 1, (b) Study area 2, (c) Study area 3.

The model was calibrated, and the passage of surge was modeled numerically with the upgraded Bay of Bengal model for estimating the surge height and water flow velocity in our analysis (S2 Fig). To capture the current condition, Cyclone Sidr (with a maximum wind speed of 248 kph, maximum wind radius of 64 km, central pressure of 928 hPa, and normal pressure of 1009 hPa) was simulated first with only the already existing polders without mangroves. To analyze the impacts of mangroves on surge height and water flow velocity, potential mangrove afforestation areas were defined and overlaid along the respective river- or sea-facing shorelines of the seven sites in the three study areas (Fig 2).

Manning’s numbers computed for the short-listed species for each study area served as estimates of water-flow resistance from the mangrove trees while passing through the forest. The model was calibrated at Hiron Point, where an automated water level gauge is maintained by the Bangladesh Inland Water Transport Authority (S3 Fig). Cyclone Sidr was simulated again with the mangrove forest. For each of the seven sites, the Sidr storm surge model was run for a range of afforestation widths and densities for the selected mangrove species (S4 Fig). The resulting effects on surge height and water flow velocity were recorded, and the best options among the range of those considered were chosen.

## Results

### Manning’s coefficients

Estimates of various mangrove species’ resistance to water flow at different planting densities and forest widths as approximated by Manning’s roughness coefficients are major contributions to the literature on nature-based disaster risk management [32,33]. Table 2 documents changes in Manning’s roughness coefficients with changes in planting density of the selected mangrove species at different water depths. As expected, the estimates confirm that resistance to water flowing through mangroves varies by mangrove species, density of trees, and water depth. For *S*. *apetala* at 7.5 m spacing, for example, the Manning’s coefficients are 8.78, 11.42, and 12.15 for water depths of 10 m, 5 m, and 2.5 m, respectively.

**Table 2:** Manning’s roughness coefficient (Measure of bed resistance) computed for root and trunk systems of selected mangrove species.

Water depth (m)	Mangrove species	Manning’s number			
		Root system	Root + trunk system (spacing)		
			**5 m**	**7.5 m**	**10 m**
10	*Sonneratia apetala*	18.24	6.27	8.78	10.78
5		16.4	8.91	11.42	12.98
2.5		13.9	10.7	12.15	12.81
			**4 m**	**6 m**	**8 m**
10	*Avicennia officinalis*	26.1	6.53	9.44	11.99
5		25.3	9.86	13.56	16.34
2.5		23.8	13.83	17.4	19.49
			**5 m**	**7 m**	**10 m**
10	*Heritiera fomes*	25.10	6.52	9.39	11.89
5		24.11	9.79	13.37	16.01
2.5		22.50	13.56	16.87	18.76
10	*Excoecaria agallocha*	24.24	7.97	11.22	13.85
5		23.14	11.59	15.17	17.51
2.5		21.41	15.08	17.78	19.12
10	*Ceriops decandra*	24.70	8.22	11.55	14.25
5		23.70	11.94	15.61	18.00
2.5		22.00	15.55	18.32	19.70

Among the selected species for this analysis, irrespective of water depth, *S*. *apetala* causes maximum obstruction, followed by *A*. *officinalis* and *H*. *fomes*. Estimates further indicate that resistance of *E*. *agallocha* exceeds that of *C*. *decandra* for all water depths considered in our analysis. For example, at 5 m water depth, the Manning’s coefficient for *S*. *apetala* is 8.91, followed by *H*. *fomes* (9.79), *A*. *officinalis* (9.86), *E*. *agallocha* (11.59), and *C*. *decandra* (11.94). As expected, resistance to water flow also increases with planting density. For example, for *S*. *apetala* at 5 m water depth, the Manning’s coefficients are 12.98, 11.42, and 8.91 at respective spacings of 10 m, 7.5 m and 5 m. However, planting density or minimum spacing between trees depends on the trunk/branch structures of the mangrove species. Field observations considering minimum distance between mature trees conclude that planting of *S*. *apetala* at 5 m spacing, *A*. *officinalis* at 4 m spacing, and *H*. *fomes*, *E*. *agallocha*, and *C*. *decandra* at 5 m spacing in the study regions are feasible and will yield the best resistance to water flow through the mangroves.

### Reduction in surge height

**Fig 3:**
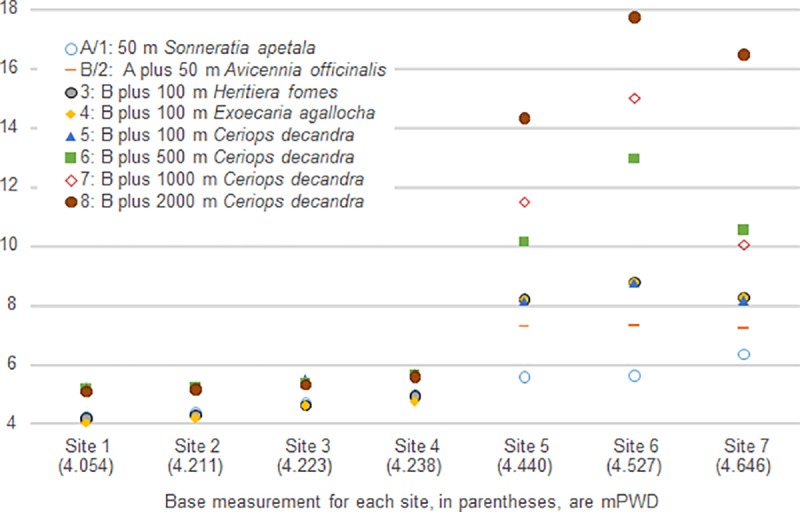
Attenuation of surge heights (cm) from afforestation of mangrove species at 5 m spacing.

Among the three study areas in our analysis, attenuation of surge height was relatively higher in study area 3, which is sea-facing and shallower compared to study areas 1 and 2, which are located on river banks. Among the seven sites considered, the reduction in surge height is in a range of 4–16.5 cm, with a median reduction of 5.5 cm (Fig 3). The least reduction in surge height, at 4 cm, was recorded at site 1 from a 50 m wide belt of *S*. *apetala* at 7.5 m spacing or a 50 m wide belt of *A*. *officinalis* at 6 m spacing. The greatest reduction in surge height, at 16.5 cm, was recorded at site 7 from a 50 m wide belt of *S*. *apetala* at 5 m spacing, followed by a 50 m wide belt of *A*. *officinalis* at 4 m spacing, and a 2 km wide belt of *C*. *decandra* at 5 m spacing (S2 Table).

### Reduction in water flow velocity

**Fig 4:**
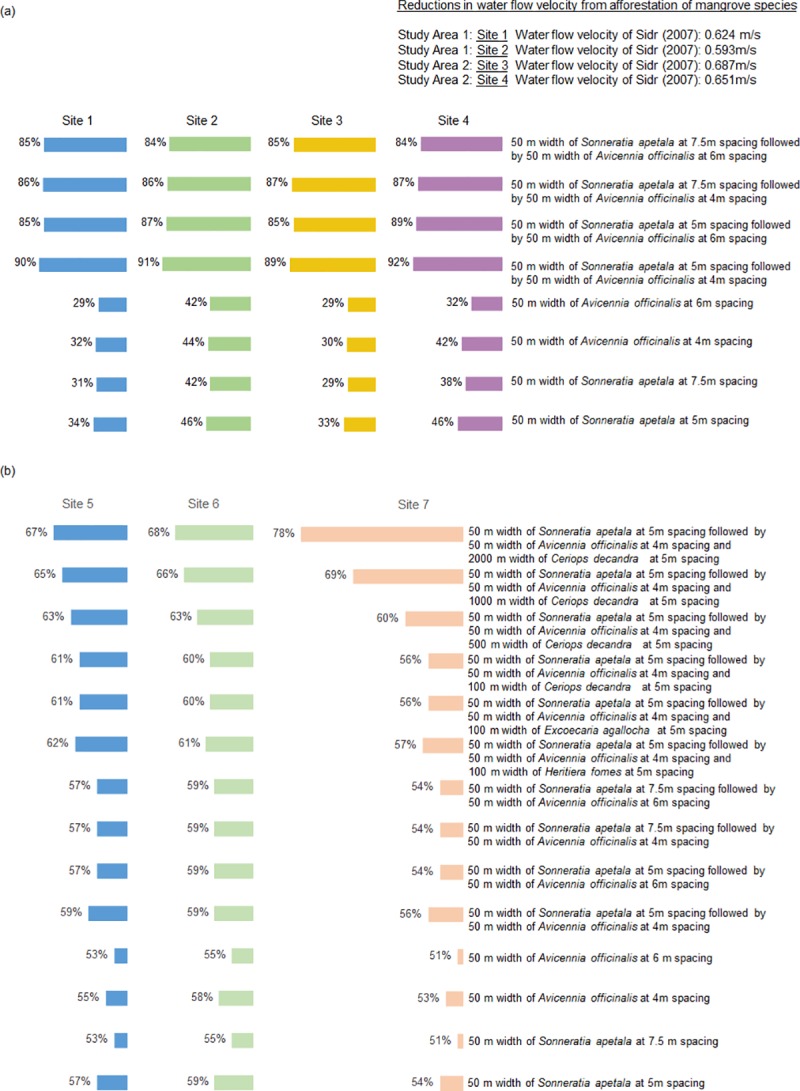
Reductions in Cyclone Sidr water flow velocity (m/s) from afforestation with mangrove species. (a) Study area 1: site 1, 0.624; site 2, 0.593. Study area 2: site 3, 0.687; site 4, 0.651. (b) Study area 3: site 5, 0.618; site 6, 0.987; site 7, 0.709.

The reduction in surge flow velocity ranged from 29% at site 1 (from a 50 m wide belt of *A*. *officinalis* at 6 m spacing) to 92% at site 4 (from a 50 m wide belt of *S*. *apetala* at 5 m spacing, followed by a 50 m wide belt of *A*. *officinalis* at 4 m spacing) (Fig 4A). The median reduction in water flow velocity, at 59 percent, was recorded at site 6 from a 50 m wide belt of *S*. *apetala* at 5 m spacing (Fig 4B).

## Discussion

The evidence that mangroves may shelter coastal communities and assets from coastal erosion, wind and swell waves, and cyclone surges is well known in tropical ecology. Some researchers skeptical about the ability of mangroves to protect against tsunamis note that mangroves might be more capable of protecting against tropical storm surges [34,35], given that surges have comparatively shorter wavelengths and more of their energy near the water surface [36]. Theoretical models indicate that mangrove forests attenuate shorter waves more than larger waves, obstructing the flow of water through the matrix of their roots, trunks, and leaves [37]. A literature review and meta-analysis of wave and storm-surge dampening by wetlands across a variety of storms and locations underscore the critical role of even narrowly-vegetated wetland sites in attenuating waves [14], as vegetation cause substantial drag [17,38]. Evidence to date further highlights that the extent of attenuation is context-dependent and exhibits nonlinear characteristics across space and time. Accordingly, additional location-specific research is necessary to design and manage these coastal forests.

### Implications for resource managers and policy makers

This study’s results confirm the efficacy of mangroves as a part of a multi-dimensional approach for protection against cyclone surges. The findings show that, for densely populated, cyclone-prone areas with a history of high storm surges, mangroves must be used together with embankments and other built infrastructure to protect assets and activities at risk. With mangroves in foreshore areas, embankments can be built lower in height, which may reduce their construction cost due to potential savings in land reclamation and earthwork. Also, by reducing water flow velocity, mangroves can reduce embankment maintenance costs by reducing toe-erosion, breaching, and other types of damages. Therefore, design parameters of embankments with foreshore areas should take the potential protection from mangroves into account. For sparsely populated rural areas, where the construction of seawalls and other hard infrastructure may not be economically feasible over long coastlines, mangroves as stand-alone natural protection against cyclone-induced surges may provide some protection.

#### Consideration of co-benefits

In addition to the impacts of mangroves on inundation during cyclones, it is also worthwhile to consider their longer-term influence on coastal dynamics, including the reduction in land erosion and accretion of foreshore area through sediment entrapment. As tidal waves move through dense forests of trunks, aerial roots, and other vegetation, water flow velocity and wave energy are impeded. As a result, the outgoing tide loses its capacity to carry away existing sediment on the mangrove forest floor, as well as sediment carried by the incoming tide, which also tends to settle on the forest floor. This process is reinforced by the presence of organic carbon materials with which sediment tends to combine. This results in vertical accretion of the forest floor with newly acquired sediment. Also, owing to the anoxic condition of the forest floor, carbon materials are not easily broken down by soil organisms. In the process, peat layers are formed and thicken over time, in turn, raising the forest floor. Roots also contribute to raising the forest floor [39].

In addition to coastal protection and cost-saving services, mangroves entrap nutrients and contaminants to maintain water quality and, compared to terrestrial forests, store a much higher amount of carbon per equivalent area [40–41]. Beyond these ecosystem services, mangroves offer a wide array of additional co-benefits, ranging from food, timber, and wood fuel to medicine and nurseries for fish and other wildlife [42–45]. Thus, to appropriately evaluate the benefits of mangrove afforestation programs, it is important to consider the multiple benefits that mangroves provide.

#### Contingency planning

Coastal-zone resource managers should note that, although mangroves can help protect against cyclone-induced storm surges, cyclones can also destroy mangroves either directly or indirectly through such effects as increased salinity or protracted anoxic conditions, which reduce mangroves’ potential for further protection. Such eventualities should be taken into account when investing in large-scale rehabilitation and replanting programs, given the long time period required for the mangrove species to reach maturity.

When designing a mangrove investment, it is also important to anticipate the stresses from a changing climate. These include rise in sea level, increase in atmospheric carbon dioxide (CO_2_), rise in air and water temperatures, changes in the frequency and intensity of precipitation and storm patterns, and progressive aquatic salinization [46]. Numerous past studies have predicted the future of the world’s mangrove forests in a changing climate; local, regional, and global forecasts range from extinction to little or no change in area coverage [47–58]. Even though mangrove loss with climate change is substantial, the potential for mangrove adaptation to sea-level rise from natural or assisted migration is also considerable. Historical evidence suggests that, with gradual sea-level rise, mangroves generally adapt [46,59]; however, with progressive aquatic salinization due to sea-level rise, the composition of mangrove species may be altered significantly [60–62]. The successful use of mangroves as part of coastal defense systems and disaster risk-reduction strategies will depend on understanding location-specific characteristics and identifying the mangrove species that can survive sea-level rise and other changes [56].

### Implications for coastal decision-makers in Bangladesh

For the densely populated, coastal region of Bangladesh, where a severe cyclone strikes every three years on average and storm surge heights are in a range of 1–4 m, mangroves alone will not fully protect assets and activities at risk. Rather, they must be integrated with embankments in coastal defense strategies. Healthy mangroves in the foreshore of embankments will contribute to savings in land reclamation and the costs of embankment rehabilitation and maintenance by protecting the built infrastructure from breaching, toe erosion, and other types of damage.

For the Coastal Embankment Improvement Project currently under way in the southwest coastal region, this means that design parameters must take into account the potential protection of mangroves in attenuating surge height and water flow velocity to complement the enhanced heightening of the selected polders. Among the mangrove species typically found in the coastal region, *S*. *apetala* causes maximum friction and hindrance to water flow, followed by *A*. *officinalis*. Both species are saline tolerant and will survive the progressive water salinization expected in a changing climate [29,30]. For embankments where foreshore area is available, even a 50–100 m wide mangrove forest with densely spaced trees will make a noticeable difference. Mangroves should be integrated into coastal defense and cyclone risk-reduction strategies in virtually all coastal settings, including river-facing and sea-facing locations in both rural and semi-urban landscapes.

Bangladesh has a long-standing mangrove afforestation program dating back to 1966. By 2013, approximately 60 km of sea-facing polders had mangrove forests in their foreshore area. Although various mangrove species were planted, a review by Siddiqi and Khan [63] pointed out that *S*. *apetala* accounted for more than 94 percent of successful mangrove plantings, while *A*. *officinalis* comprised less than 5 percent. *S*. *apetala* performed particularly well along the coastline, while the success of *A*. *officinalis* was limited mainly to the eastern coastal zone [64].

This historical evidence is promising since, among the five species considered in this analysis, *S*. *apetala* showed maximum potential for attenuation of storm surge and water flow velocity. At maturity, *S*. *apetala* can reach up to 20 m; this species is also effective at blocking erosion and quickening land accretion. But experience suggests that areas afforested with only *S*. *apetala* are susceptible to pest attacks. Thus, a diverse mix of mangrove species would reduce the risk of pest infestation. Since *S*. *apetala* colonizes only on mud flats in newly emergent areas where sedimentation is still ongoing, the only other candidate mangrove species for companion planting are *A*. *officinalis* and *B*. *gymnorhiza*. Because the survival rates for both *S*. *apetala* and *A*. *officinalis* are less than 50% after five years, one should plan for replacement plantings after three years. By that time, with vertical accretion, the land will be unsuitable for replanting with *S*. *apetala*. For replacement planting, *C*. *decandra*, *E*. *agallocha*, and *H*. *fomes* are suitable species for a permanent forest cover.

## Supporting information

S1 Fig Field measurements of the trunk system of *Sonneratia apetala* (left) and the roots of *Avicennia officinalis* (right).
(TIF)

S2 Fig Geographical extent of the Bay of Bengal model used in this analysis. (TIF)

S3 Fig Storm surge calibration at Hiron Point, Cyclone Sidr. (TIF)

S4 Fig Effect of afforestation width on surge height and velocity for site 3. (TIF)

S1 Table Field data on characteristics of the mangrove species analyzed. (DOCX)

S2 Table Attenuation of surge heights from afforestation of mangrove species: Study areas 1–3. (DOCX)
