# Supplementary figures and images for: Quantifying the protective capacity of mangroves from storm surges in coastal Bangladesh
Source: PLoS One. 2019 Mar 21;14(3):e0214079. doi: 10.1371/journal.pone.0214079 (PMC6428389; doi:10.1371/journal.pone.0214079)

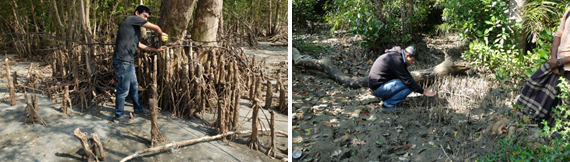

Supplement: S1 Fig — Field measurements of the trunk system of Sonneratia apetala (left) and the roots of Avicennia officinalis (right). (TIF) [file pone.0214079.s001.tif]

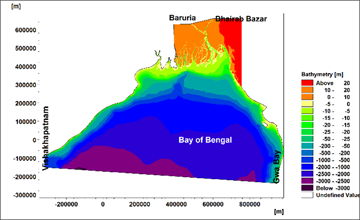

Supplement: S2 Fig — (TIF) [file pone.0214079.s002.tif]

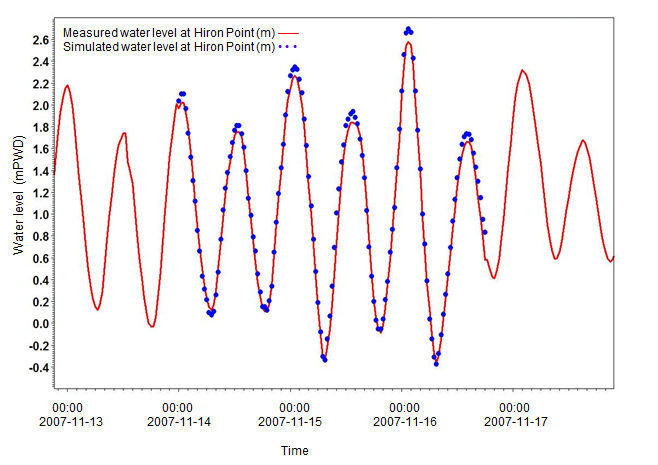

Supplement: S3 Fig — (TIF) [file pone.0214079.s003.tif]

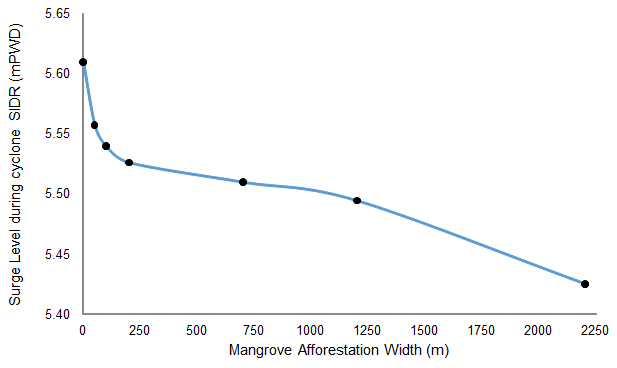

Supplement: S4 Fig — (TIF) [file pone.0214079.s004.tif]
