# Supplementary material for: Quantifying the protective capacity of mangroves from storm surges in coastal Bangladesh
Source: PLoS One. 2019 Mar 21;14(3):e0214079. doi: 10.1371/journal.pone.0214079 (PMC6428389; doi:10.1371/journal.pone.0214079)
Supplement: S1 Table — (DOCX) [file pone.0214079.s005.docx]

| **Mangrove species** | **Trunk diameter (m)** | **Root diameter (m)** | **Root height from existing ground level (m)** |
| --- | --- | --- | --- |
| *Sonneratia apetala* | 0.51 | 0.15 | 1.04 |
| *Avicennia officinalis* | 0.32 | 0.05 | 0.23 |
| *Heritiera fomes* | 0.5 | 0.05 | 0.28 |
| *Excoecaria agallocha* | 0.3 | 0.08 | 0.33 |
| *Ceriops decandra* | 0.3 | 0.05 | 0.3 |

**S1 Table. Field data on characteristics of the mangrove species analyzed.**
