# Supplementary material for: Quantifying the protective capacity of mangroves from storm surges in coastal Bangladesh
Source: PLoS One. 2019 Mar 21;14(3):e0214079. doi: 10.1371/journal.pone.0214079 (PMC6428389; doi:10.1371/journal.pone.0214079)
Supplement: S2 Table — (DOCX) [file pone.0214079.s006.docx]

| **Scenario** | **Site 1** | | **Site 2** | |
| --- | --- | --- | --- | --- |
|  | **Surge level**  **(m PWD)** | **Attenuation of surge height from baseline (cm)** | **Surge level**  **(m PWD)** | **Attenuation of surge height from baseline (cm)** |
| Baseline (without mangroves) | 4.054 |  | 4.211 |  |
| 50 m *Sonneratia apetala*, 5m spacing | 4.011 | 4.223 | 4.168 | 4.367 |
| 50 m *S. apetala*, 7.5 m spacing | 4.013 | 4.033 | 4.169 | 4.204 |
| 50 m *Avicennia officinalis*, 4m spacing | 4.012 | 4.164 | 4.168 | 4.304 |
| 50 m *A. officinalis*, 6 m spacing | 4.013 | 4.014 | 4.170 | 4.184 |
| 50 m *S. apetala*, 5 m spacing, followed by 50 m *A. officinalis*, 4 m spacing | 4.001 | 5.233 | 4.159 | 5.245 |
| 50 m *S. apetala*, 5 m spacing, followed by 50 m *A. officinalis*, 6 m spacing | 4.002 | 5.151 | 4.159 | 5.219 |
| 50 m *S. apetala*, 7.5 m spacing, followed by 50 m *A. officinalis*, 4 m spacing | 4.002 | 5.140 | 4.160 | 5.177 |
| 50 m *S. apetala*, 7.5 m spacing, followed by 50 m *A. officinalis*, 6 m spacing | 4.003 | 5.100 | 4.160 | 5.150 |

**S2a Table. Attenuation of surge heights from afforestation of mangrove species: Study area 1.**

| **Scenario** | **Site 3** | | **Site 4** | |
| --- | --- | --- | --- | --- |
|  | **Surge level (m PWD)** | **Attenuation of surge height from baseline (cm)** | **Surge level (m PWD)** | **Attenuation of surge height from baseline (cm)** |
| Baseline (without mangroves) | 4.223 |  | 4.238 |  |
| 50 m *Sonneratia apetala*, 5 m spacing | 4.176 | 4.677 | 4.188 | 4.981 |
| 50 m *S. apetala*, 7.5 m spacing | 4.177 | 4.603 | 4.189 | 4.889 |
| 50 m *Avicennia officinalis*, 4 m spacing | 4.177 | 4.619 | 4.189 | 4.912 |
| 50 m *A. officinalis*, 6 m spacing | 4.177 | 4.589 | 4.191 | 4.739 |
| 50 m *S. apeta*, 5 m spacing, followed by 50 m *A. officinalis*, 4 m spacing | 4.168 | 5.484 | 4.181 | 5.696 |
| 50 m *S. apetala*, 5 m spacing, followed by 50 m *A. officinalis*, 6 m spacing | 4.169 | 5.359 | 4.182 | 5.652 |
| 50 m *S. apetala*, 7.5 m spacing, followed by 50 m *A. officinalis*, 4 m spacing | 4.170 | 5.330 | 4.182 | 5.624 |
| 50 m *S. apetala*, 7.5 m spacing, followed by 50 m *A. officinalis*, 6 m spacing | 4.170 | 5.320 | 4.182 | 5.584 |

**S2b Table. Attenuation of surge heights from afforestation of mangrove species: Study area 2.**

| **Scenario (5 m spacing)** | **Site 5** | | **Site 6** | | **Site 7** | |
| --- | --- | --- | --- | --- | --- | --- |
|  | **Surge level (m PWD)** | **Attenuation of surge height from baseline (cm)** | **Surge level (m PWD)** | **Attenuation of surge height from baseline (cm)** | **Surge level (m PWD)** | **Attenuation of surge height from baseline (cm)** |
| Baseline (without mangroves) | 4.440 |  | 4.527 |  | 4.646 |  |
| 50 m *Sonneratia apetala* | 4.384 | 5.588 | 4.471 | 5.620 | 4.583 | 6.347 |
| 50 m *S. apetala*, plus 50 m *Avicennia officinalis* | 4.367 | 7.282 | 4.454 | 7.308 | 4.574 | 7.214 |
| 50 m *S. apetala*, plus 50 m *A. officinalis* and 100 m *Heritiera fomes* | 4.358 | 8.203 | 4.440 | 8.776 | 4.564 | 8.260 |
| 50 m *S. apetala*, plus 50 m *A. officinalis* and 100 m *Excoecaria agallocha* | 4.358 | 8.188 | 4.440 | 8.759 | 4.564 | 8.250 |
| 50 m *S. apetala*, plus 50 m *A. officinalis* and 100 m *Ceriops decandra* | 4.359 | 8.127 | 4.440 | 8.746 | 4.565 | 8.146 |
| 50 m *S. apetala*, plus 50 m *A. officinalis* and 500 m *C. decandra* | 4.339 | 10.130 | 4.398 | 12.948 | 4.541 | 10.527 |
| 50 m *S. apetala*, plus 50 m *A. officinalis* and 1000 m *C. decandra* | 4.325 | 11.483 | 4.377 | 15.000 | 4.546 | 10.056 |
| 50 m *S. apetala*, plus 50 m *A.* *officinalis* and 2000 m *C. decandra* | 4.297 | 14.329 | 4.350 | 17.752 | 4.481 | 16.476 |

**S2c Table. Attenuation of surge heights from afforestation of mangrove species: Study area 3.**
